# The demographic and life‐history costs of fear: Trait‐mediated effects of threat of predation on *Aedes triseriatus*

**DOI:** 10.1002/ece3.5003

**Published:** 2019-03-01

**Authors:** Geoffrey D. Ower, Steven A. Juliano

**Affiliations:** ^1^ School of Biological Sciences Illinois State University Normal Illinois; ^2^ Illinois Natural History Survey Prairie Research Institute University of Illinois at Urbana‐Champaign Champaign Illinois

**Keywords:** carryover effects, chemical cues, life‐history traits, predation, trade‐offs, trait‐mediated effects

## Abstract

1. Predators alter prey populations via direct lethality (density‐mediated effects), but in many taxa, the indirect nonlethal threat of predation may be almost as strong an effect, altering phenotypically plastic traits such as prey morphology, behavior, and life history (trait‐mediated effects). There are costs to antipredator defenses and the strength of prey responses to cues of predation likely depends on both the perceived level of risk and food availability.
2. The goal of this study was to test the hypothesis that the costs of nonlethal trait‐mediated interactions impacting larvae can have carryover effects that alter life‐history traits, adult characteristics, and ultimately population dynamics.
3. The effects of *Toxorhynchites rutilus* kairomones and chemical alarm cues on *Aedes triseriatus* were assessed in a two‐level factorial design manipulating nutrient level (low or high) and chemical cues of predation (present or absent).
4. Nonlethal chemical cues of predation significantly decreased female survivorship and significantly decreased female size. Females emerged as adults significantly earlier when exposed to predation cues when there was high nutrient availability. When raised in the low nutrient treatment and exposed to predator cues, adult females had 2.1 times the hazard of death compared to high nutrient‐no predator cues. Females raised in the high nutrient and predator cue treatment blood fed sooner than did females from other combinations.
5. Fear of predation can substantially alter prey life‐history traits and behavior, which can cascade into dramatic population, community, and ecosystem effects. Exposure to predator cues significantly decreased the estimated cohort rate of increase, potentially altering the expected population density of the next generation.

## 1 INTRODUCTION

Predation is a major selective force that shapes prey populations in a wide number of taxa including planktonic protists (Harvey, Jeong, & Menden‐Deuer, 2013), fish (Ruell et al., 2013), arachnids (Persons, Walker, Rypstra, & Marshall, 2001), insects (Ball & Baker, 1996; Kesavaraju, Alto, Lounibos, & Juliano, 2007; Kesavaraju & Juliano, 2010; McCauley, Rowe, & Fortin, 2011; Peckarsky & McIntosh, 1998), anurans (Skelly & Werner, 1990), mammals (Apfelbach, Blanchard, Blanchard, Hayes, & McGregor, 2005), and birds (Dorset, Sakaluk, & Thompson, 2017; Roth, Cox, & Lima, 2008). Predator–prey arms races favor predators with enhanced prey detection and capture traits, which may intensify selection on prey to improve predator detection and avoidance. Prey can detect predators via kairomones, disturbance cues released by startled prey (e.g., urine), alarm cues released by injured conspecifics or heterospecifics, and diet cues from digestion of and defecation of the prey (Ferrari, Wisenden, & Chivers, 2010; Wisenden, 2000, 2003).

In addition to directly affecting prey fitness and populations via lethal density‐mediated effects, predators also indirectly affect prey by inducing phenotypically plastic morphological defences, altering prey foraging behaviors, and changing prey rates of growth and development through life‐history stages (Benard, 2004; Wisenden, 2003). Meta‐analysis showed that these trait‐mediated effects can be just as strong as the density‐mediated effect of lethality (Preisser, Bolnick, & Benard, 2005). These trait‐mediated effects are varied and often context‐dependent. The phytoplanktonic flagellate *Phaeocystis globosa* respond to predator cues from grazing ciliates by aggregating into large colonies up to 30,000 μm in diameter that are less vulnerable to ciliate predators, but exposure to copepod predator cues suppresses colony formation by 60%–90% because copepods prey upon the large colonies (Long, Smalley, Barsby, Anderson, & Hay, 2007). Salamanders exposed to predator cues from garter snakes reduced foraging activity to decrease their predation risk (Maerz, Panebianco, & Madison, 2001). Exposure of a mouthbrooding cichlid (*Eretmodus cyanostictus*) to visual and chemical cues of predation resulted in larger eggs and hatchlings, and this response is likely adaptive because predation risk substantially decreases for fish larvae as they increase in size (Segers & Taborsky, 2012). The live‐bearing guppy (*Poecilia reticulata*) has impaired swimming abilities during late‐term pregnancy and reduces their predation risk by shortening brood retention times when exposed to predator cues (Evans, Gasparini, & Pilastro, 2007).

The observed phenotypic plasticity in prey responses to predation can be adaptive because there are trade‐offs associated with morphological, behavioral, and life‐history antipredator defenses (Anholt & Werner, 1999). Reducing foraging activity to decrease predation risk comes at the expense of increased starvation risk or slower growth. Prey in low nutrient environments may not be able to afford to decrease foraging to reduce their predation risk. Maturing sooner to escape a high predation habitat may come at the expense of reproductive fitness, such as reduced sexual attractiveness (Ruell et al., 2013) or fecundity (Ball & Baker, 1996). These trade‐offs mean that plastic responses are advantageous when predator presence or abundance is variable.

Larval mosquitoes are a good system for experimental studies of the trade‐offs associated with antipredator defenses. Although intensity of predation is strongest for mosquito larvae that develop in permanent bodies of water (Juliano, 2007; Washburn, 1995), larval mosquitoes that develop in ephemeral water‐filled natural and manmade containers also are frequently exposed to predators that specialize on those habitats, and may show strong trait‐based responses to those predators (e.g., Bellamy & Alto, 2018; Chandrasegaran, Kandregula, Quader, & Juliano, 2018; Costanzo, Muturi, & Alto, 2011; Kesavaraju & Juliano, 2004; van Uitregt, Hurst, & Wilson, 2012). Small containers and short generation times make it possible to run replicated experiments of generation‐length duration. *Toxorhynchites* is a unique genus of mosquitoes that are container‐dwelling ambush predators as larvae, feeding primarily on other mosquito larvae and pupae (Steffan & Evenhuis, 1981). The North American tree‐hole mosquito *Aedes triseriatus* frequently co‐occurs as larvae with *Toxorhynchites rutilus*. Larval *A. triseriatus* feed on microorganisms growing on decaying plant and animal detritus (Daugherty, Alto, & Juliano, 2000; Merritt, Dadd, & Walker, 1992).

In response to alarm cues from predation, *A. triseriatus* reduce their predation risk via a threat‐sensitive reduction in foraging behavior and by avoiding the container bottom where *T. rutilus* frequently ambush prey (Costanzo et al., 2011; Hechtel & Juliano, 1997; Juliano & Gravel, 2002; Kesavaraju, Damal, & Juliano, 2007; Kesavaraju & Juliano, 2004; Wormington & Juliano, 2014). These kinds of changes in behavior are likely to have life‐history costs with carryover effects on adult traits such as body size, time to adulthood, adult longevity, and blood feeding (O'Connor, Norris, Crossin, & Cooke, 2014). Cues from a predator induced both altered larval behavior and prolonged time to adulthood in *A. triseriatus* (Costanzo et al., 2011). Because the indirect effects of threat of predation are likely mediated through modifications of the energy budget, we expect that the effects of threat of predation should be more severe when resources for larvae are scarce and should impact females, which are more sensitive to reduced resource acquisition (Chandrasegaran et al., 2018). To understand the consequences of threat of predation at the population level, comprehensive investigation of context‐dependent impacts on life‐table variables (survivorship, age at reproduction, adult feeding success, and fecundity) is necessary (Chandrasegaran et al., 2018). Because *A. triseriatus* exhibits greater behavioral responses to predation threat than other container *Aedes* (Costanzo et al., 2011; Grill & Juliano, 1996; Kesavaraju & Juliano, 2004), *A. triseriatus* is the ideal focal species for investigating potential life‐history responses in fecundity, longevity, biting rates, and population dynamics. Any impact of predators on populations of *A. triseriatus* is likely of some practical importance because *A. triseriatus* are the primary vector of La Crosse virus, which annually causes an average of 63 human cases of encephalitis and 1 death in the United States (CDC, 2017; Eldridge, Scott, Day, & Tabachnick, 2004). The goal of this study was to test the hypothesis that costly nonlethal trait‐mediated effects of predator cues on *A. triseriatus* affect traits of adults, life‐history traits, and ultimately population dynamics.

## 2 MATERIALS AND METHODS

Experimental *T. rutilus* predators and *A. triseriatus* prey were from laboratory colonies established during the summer of 2016 with field‐collected larvae from Washington University Tyson Research Center located 30 km southwest of St. Louis, Missouri, USA (38.5187002, −90.5567767). Individuals used in the experiments likely were 2–4 generations removed from field collection. All mosquitoes in the colonies and experiments were reared at 25°C on a 14:10 light:dark cycle. The *A. triseriatus* eggs were synchronously hatched by submerging oviposition papers in 0.4 g/L nutrient broth in 15‐ml glass vials. Hatched 1st instar larvae were counted into cohorts of 100 prey with 7 replicates for each two‐factor combination of nutrient (low or high) and predator cue (present or absent) treatments.

### 2.1 Nutrient treatments

To allow sufficient time for bacterial growth, experimental containers were set‐up 4 days prior to hatching the *A. triseriatus* larvae by adding oven‐dried (50°C) white oak (*Quercus alba*) leaves and decorated crickets (*Gryllodes sigillatus*) to 470 ml of reverse osmosis (RO) water in 500‐ml opaque plastic containers. Crickets were ground into a powder prior to weighing. The low nutrient containers received 0.5 g oak leaves and 0.0375 g crickets, and the high nutrient containers received 1.0 g oak leaves and 0.075 g crickets. To simulate additional detritus inputs, every 10th day an additional 0.0375 and 0.075 g of dried crickets were added to the low and high nutrient treatment containers, respectively.

### 2.2 Predator cue treatments

The nonlethal effects of predation were explored by exposing *A. triseriatus* larvae to chemical cues of predation using predator enclosures suspended in the larger container housing detritus and experimental larvae (Chandrasegaran et al., 2018). Ten conspecific 4th instar prey were placed inside each predator enclosure for both control and nonlethal treatments and were replenished daily if they were eaten, died, or pupated. A 4th instar *T. rutilus* was placed inside the predator enclosures of the nonlethal predator cue treatments. To provide a continuous predator cue, 4th instar *T. rutilus *that pupated or died were replaced by another 4th instar predator. Prey body parts and corpses were left in the predator enclosure because they are known to be an important component of the predation alarm cues for *A. triseriatus* (Kesavaraju & Juliano, 2010).

Predator enclosures were 3.5‐cm‐diameter PVC pipe cut into 10 cm sections (Chandrasegaran et al., 2018). Two 4‐mm‐diameter holes were drilled 4 cm from the tops and these were used to suspend the predator enclosures in the containers with bamboo skewers. Fine (0.3 mm opening) and coarse (0.6 mm opening) nylon netting was affixed to the bottoms of the PVC pipe using rubber bands and secured with a zip ties. Netting allowed predator cues to diffuse from the enclosure but ensured that larvae could not get in or out. The outer fine netting was removed after focal *A. triseriatus* larvae had all reached 3rd instar, because 3rd and 4th instars were too large to fit through the coarse netting, but the coarse netting allowed small pieces of victims or predator feces to fall through the mesh, providing a stronger and more realistic predator cue to the focal *A. triseriatus* prey.

### 2.3 Adult longevity, fecundity, and population rate of increase

Containers were checked daily, and when focal *A. triseriatus* pupated, they were isolated into 15‐ml vials with nylon netting affixed over the top with a rubber band. Each female was randomly assigned to either fecundity/longevity or energy reserve/longevity assays. All males were assigned to energy reserve/longevity assays.

Females in the fecundity/longevity assays were provided with cotton soaked in 20% sucrose, which was rewetted daily. On day 4, females were deprived of sugar water for 12 hr prior to blood feeding them with mice anesthetized with ketamine–xylazine and placed on top of the vial netting (IACUC protocol #842043). Each *A. triseriatus* female was given at least 2 opportunities to blood feed. The amount of blood that each female ingested was not quantified, but females were allowed to feed to repletion. Ten days after blood feeding females were frozen, and their ovaries were later dissected and mature eggs were counted. Wing measurements were taken for only a subset of the females in the fecundity/longevity trials (107 out of 336 total), because most of their wings were too badly damaged by flying in confinement, precluding accurate measurement. We acknowledge that only using undamaged wings could result in a biased sample, particularly because females that die sooner might have less wing damage. However, these were the best data we could collect because it is difficult to cage females for several days without wing damage occurring from female flight. Mosquitoes still attempt to fly even in small vials.

Females and males in the energy reserve/longevity assays were provided with cotton soaked in RO water, which was rewetted daily. Each individual was checked every 8 hr, and their time of death was recorded.

Wing measurements were taken for all females with undamaged wings, which positively correlate with fecundity in many species of mosquitoes (e.g., Briegel, 1990). Wing lengths were used to estimate the population rate of increase using the composite index of performance, which is an analogous approach to calculating population growth rates using life tables (Livdahl & Sugihara, 1984):

(1) $$r^{\prime }=\frac{\ln(\frac{1}{N_{0}}\sum_{x}A_{x}f\left(\bar{w_{x}}\right))}{D+\;\frac{\sum_{x}xA_{x}f\left(\bar{w_{x}}\right)}{\sum_{x}A_{x}f\left(\bar{w_{x}}\right)}},$$

where *D* is the mean time from female eclosion and oviposition, which was estimated to be 12 days (Aspbury & Juliano, 1998; Léonard & Juliano, 1995), *A_x_* is the number of females eclosing from a container on day *x*, and $f(\bar{w_{x}})$is the predicted number of female eggs laid, which was estimated from the mean wing lengths of females eclosing on day *x* using the equation $f\left(\bar{w_{x}}\right)=\;23.17\bar{w_{x}}\;-\;51.1$ (Livdahl, 1984). These measurements are combined in a form analogous to the life‐table calculation of per capita rate of change for a population as estimated by the natural log of the net reproductive rate divided by the cohort generation time: *r = ln*(*R_0_*)/*T_c _*(Livdahl & Sugihara, 1984). In this index, *A_x_*/*N_0_* substitutes for *l_x_*, fecundity predicted from wing length substitutes for *m_x_*, and the denominator of Equation ((1)) substitutes for *T_c_* (Livdahl & Sugihara, 1984). The composite index *r′ *uses cohort data on the number of females surviving to adulthood and makes the assumption that each female lives long enough to find one blood meal and produce one batch of eggs, that mean wing length is an accurate predictor of number of eggs produced, and that age at first reproduction is a fixed number of days after eclosion (Livdahl & Sugihara, 1984). These simplifying assumptions have been tested in two laboratory studies comparing the index *r′ *to true life‐table estimates of *r *(Chandresegaran & Juliano, 2019; Chmielewski, Khatchikian, & Livdahl, 2010). Both studies found that *r′* was a significant predictor of *r* with Pearson correlation coefficients of 0.5–0.8.

### 2.4 Statistical analyses

Proportion of female and male *A. triseriatus* surviving to adulthood for replicate cohorts was tested with two‐way analyses of variance (ANOVA) with predator cue, nutrient level, and their interaction as fixed effects (PROC GLM, SAS 9.4). Sex could not be determined for dead larvae, so a 1:1 sex ratio was assumed in calculating survivorship. Wing length of females was analyzed with a two‐way mixed model ANOVA, with predator cue, nutrient level, and their interaction as fixed effects, and container nested in nutrient–predator cue combination (i.e., replicate cohort) as a random effect that was used as the denominator for all *F* tests (PROC MIXED, SAS 9.4).

Time from hatch to female eclosion as adults and times from eclosion as adult until adult female death in energy reserve/longevity assays were analyzed with Cox proportional hazards models with the wing lengths, nutrient and predator treatments, and their interaction as fixed effects, individual container (=experimental unit) as a random effect, and *df* adjusted for the presence of the random effect (PROC PHREG, SAS 9.4). Longevity (eclosion as adult to death) for females provided with 20% sucrose was analyzed separately for each nutrient treatment, because substantially more values were censored in the high nutrient treatments due to a greater proportion of females blood feeding (see Results) and therefore being frozen on day 10 for ovary dissections. Wing length was also dropped as a factor in the fecundity/longevity analyses because most wings were too battered to measure. A fixed‐effects generalized linear model (PROC GLIMMIX, SAS 9.4) with a binomial distribution and logit link function was fit to the proportion of females that blood fed for each nutrient by predator treatment combination. A Cox proportional hazards analysis was also used to analyze female time from eclosion as adult to blood feeding with nutrient and predator treatments and their interaction as fixed effects and individual container (=experimental unit) as a random effect. Females that would not blood feed were censored at the last time they were offered an opportunity to blood feed. “Hazard ratios” were estimated, which describe the relative “risk” of an event occurring (e.g., adult emergence, adult death, and blood feeding) for an individual in the nutrient and predator treatments (SAS, 2017). For categorical variables, a hazard ratio indicates elevated risk, relative to a reference category, if it is significantly greater than 1 or reduced risk if it is significantly less than 1. For continuous variables (e.g., wing length), hazard ratios estimate increase or decrease in risk for a unit increase in the continuous variable.

Path analysis was used to test for direct and indirect effects of nutrient and predator cues on measured wing lengths and fecundity as quantified by counting eggs in dissected females (PROC CALIS, SAS 9.4). Females whose fecundity was not quantified by dissection were not included in the path analysis. This analysis is important for determining the mechanisms producing any effects of larval nutrition or predation cues for larvae on fecundity. Indirect effects on fecundity would arise if manipulated variables (larval nutrition, predation cues to larvae) affect size of the adult female, which in turn affects fecundity. Direct effects of manipulated variables would arise if manipulated variables affect fecundity through mechanisms that do not affect adult size (e.g., energy or nutrient storage and increased number of ovarioles). In path analysis, the same variable can be both independent and dependent for other variables in the model (Mitchell, 2001; Wright, 1921). Path coefficients quantify the direct effect that variance of the independent variables has on dependent variables, independent of the direct effects from other independent variables (Yee, Kaufman, & Juliano, 2007). The full model was compared with two reduced models, one in which we postulated the absence of direct effects nutrient and predator cues on fecundity, and a second in which we additionally postulated no effect of predator cues on wing length. Akaike's information criterion was used to select the best model.

## 3 RESULTS

### 3.1 Survivorship

**Figure 1:**
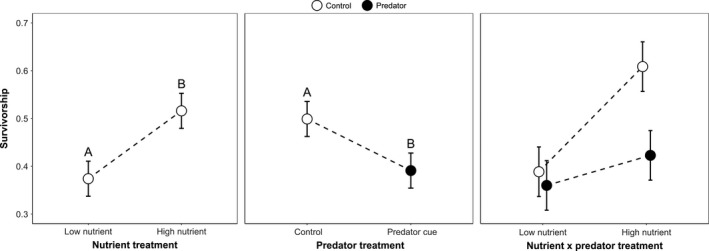
Least squares mean survivorship ± *SE* was significantly greater in the high versus low nutrient treatment, and in the control versus predator cue treatment, based on Tukey–Kramer multiple comparisons (indicated by the letters above the means). Pairwise comparisons were not done for the nonsignificant interaction term

Female survivorship to adulthood was significantly decreased when exposed to predator cues, significantly increased by greater nutrient availability, and unaffected by the interaction (ANOVA: Predator: *F*_1,24_ = 4.27, *p* = 0.0499; Nutrient: *F*_1,24_ = 7.43, *p* = 0.0118; Predator × Nutrient: *F*_1,24_ = 2.29, *p* = 0.143; Figure 1). Male survivorship significantly increased with greater nutrient availability but was not significantly affected by predator cues or the interaction between nutrient and predator cue treatments (ANOVA: Predator: *F*_1,24_ = 1.55, *p* = 0.225; Nutrient: *F*_1,24_ = 8.84, *p* = 0.0066; Predator × Nutrient: *F*_1,24_ = 2.1, *p* = 0.161).

### 3.2 Female size

**Figure 2:**
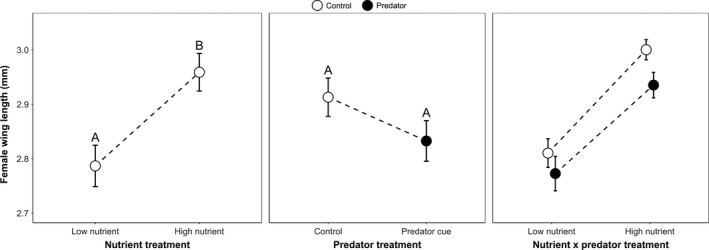
Least squares mean female wing lengths ± SE was significantly greater in the high versus low nutrient treatment, based on Tukey–Kramer multiple comparisons (indicated by the letters above the means). Pairwise comparisons were not done for the nonsignificant interaction term

High nutrients yielded significantly greater wing lengths than low nutrients, but exposure to predator cues and predator X nutrient interaction had no significant effects (ANOVA: Nutrient: *F*_1,24_ = 11.24, *p* = 0.0026; Predator: *F*_1,24_ = 2.46, *p* = 0.1301; Predator × Nutrient: *F*_1,24_ = 0.03, *p* = 0.8735; Figure 2).

### 3.3 Emergence

**Figure 3:**
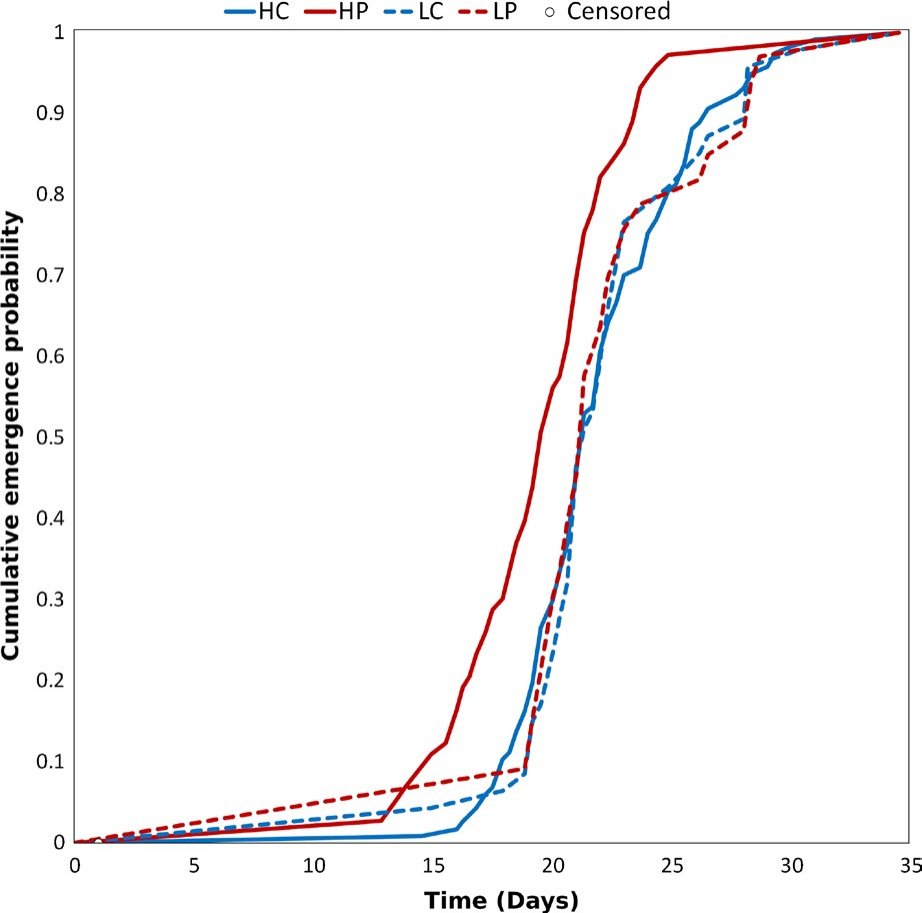
Probability of female adult emergence over time. There were no censored points

Female adult emergence time depended upon the interaction between predator cue and nutrient treatments (Cox proportional hazard model: Predator: Wald χ^2^ = 0.0002, adjusted *df *= 0.54, *p* = 0.905; Nutrient: Wald χ^2^ = 6.505, adjusted *df *= 0.52, *p* = 0.004; Predator × Nutrient: Wald χ^2^ = 2.895, adjusted *df *= 0.48, *p* = 0.034; Wing length: Wald χ^2^ = 0.021, adjusted *df *= 0.92, *p* = 0.859; Container: Wald χ^2^ = 24.956, adjusted *df *= 11.26, *p* = 0.011; Figure 3). Females in the high nutrient treatment that were exposed to predator cues had 2.1 times greater hazard of emergence (i.e., were more likely to emerge as adults sooner) compared with females in the high nutrient treatment not exposed to predator cues, whereas females in the low nutrient treatments had approximately the same hazard of emergence irrespective of predator cue exposure.

The predator cue treatment and its interaction with the nutrient treatment had no significant effects on male emergence time, but males had 1.7 times greater hazard of emergence if they were in the high nutrient treatment (Cox regression: Predator: Wald χ^2^ = 0.000, adjusted *df *= 0.18, *p* = 0.632; Nutrient: Wald χ^2^ = 4.185, adjusted *df *= 0.16, *p* = 0.004; Predator × Nutrient: Wald χ^2^ = 0.026, adjusted *df *= 0.16, *p* = 0.269; Container: Wald χ^2^ = 123.337, adjusted *df* = 20.05, *p* < 0.0001).

### 3.4 Adult longevity

#### 3.4.1 Nutrient reserve/longevity assays

**Figure 4:**
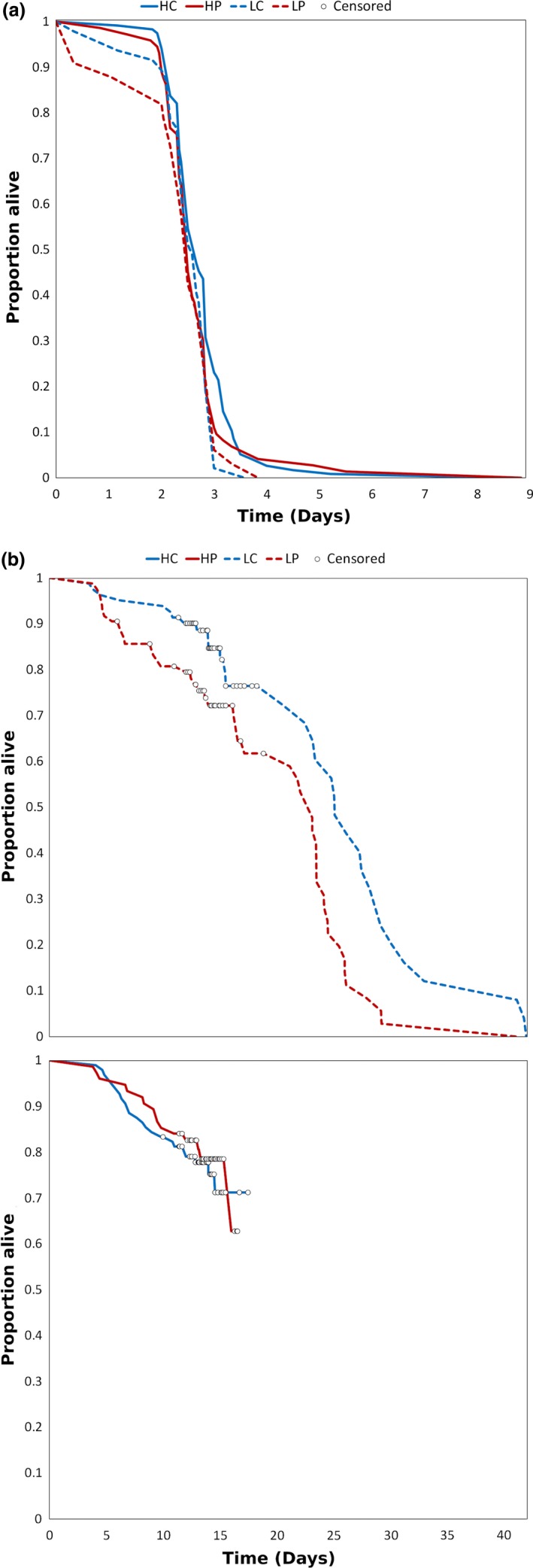
Survival probability of female adults in the (a) energy reserves/longevity assays with water only, and (b) fecundity/longevity assays with sugar water. Censored points are females that were frozen after day 10 to for ovary dissections and wing length measurement. Females in the low and high nutrient treatments were analyzed separately for fecundity/longevity assays due to having a different number of censored observations

The longevity of *A. triseriatus* females provided only with water was significantly affected by wing length and not by predator cue, nutrient treatment, or their interaction (Figure 4a, Cox regression: Predator: Wald χ^2^ = 0.047, adjusted *df *= 0.72, *p* = 0.71; Nutrient: Wald χ^2^ = 0.019, adjusted *df *= 0.72, *p* = 0.789; Predator × Nutrient: Wald χ^2^ = 0.003, adjusted *df *= 0.68, *p* = 0.882; Wing length: Wald χ^2^ = 14.757, adjusted *df *= 0.90, *p* ≤ 0.0001; Container: Wald χ^2^ = 9.619, adjusted *df *= 6.47, *p* = 0.173). Each 1 mm decrease in female wing size increased their hazard of death by 3.1 times. Because wing length was significantly affected by nutrient treatment (see above), we also tested alternative models omitting either wing length or nutrient treatment (and interactions), to determine whether the correlation of those two independent variables affected conclusions about significant effects. All alternative models yielded no significant effect of nutrients and a significant effect of wing length, and AIC was least for the model reported above (results not shown).

There was no effect of nutrient or predator cue treatments on longevity of male *A. triseriatus* adults (Cox regression: Predator: Wald χ^2^ = 2.718, adjusted *df* = 0.62, *p* = 0.0529; Nutrient: Wald χ^2^ = 0.552, adjusted *df* = 0.58, *p* = 0.277; Predator × Nutrient: Wald χ^2^ = 0.295, adjusted *df* = 0.59, *p* = 0.386; Container: Wald χ^2^ = 16.166, adjusted *df* = 9.60, *p* = 0.0813).

#### 3.4.2 Fecundity/longevity assays

There was a significant difference between predator treatments in the longevity of females provided with 20% sucrose solution in low nutrient containers (Figure 4b, Cox regression: Predator: Wald χ^2^ = 9.164, adjusted *df *= 1.0, *p* = 0.003). The hazard ratio for females in the low nutrient treatments was two times greater (i.e., they died sooner) if they had been exposed to predator cues compared to females in the low nutrient treatment that were not exposed to predator cues during larval development. In high nutrient containers, there was no significant difference between predator treatments in the longevity of female adults provided with 20% sucrose (Figure 4b, Cox regression: Predator: Wald χ^2^ = 0.066, adjusted *df *= 1.0, *p* = 0.797).

### 3.5 Willingness to blood feed

**Figure 5:**
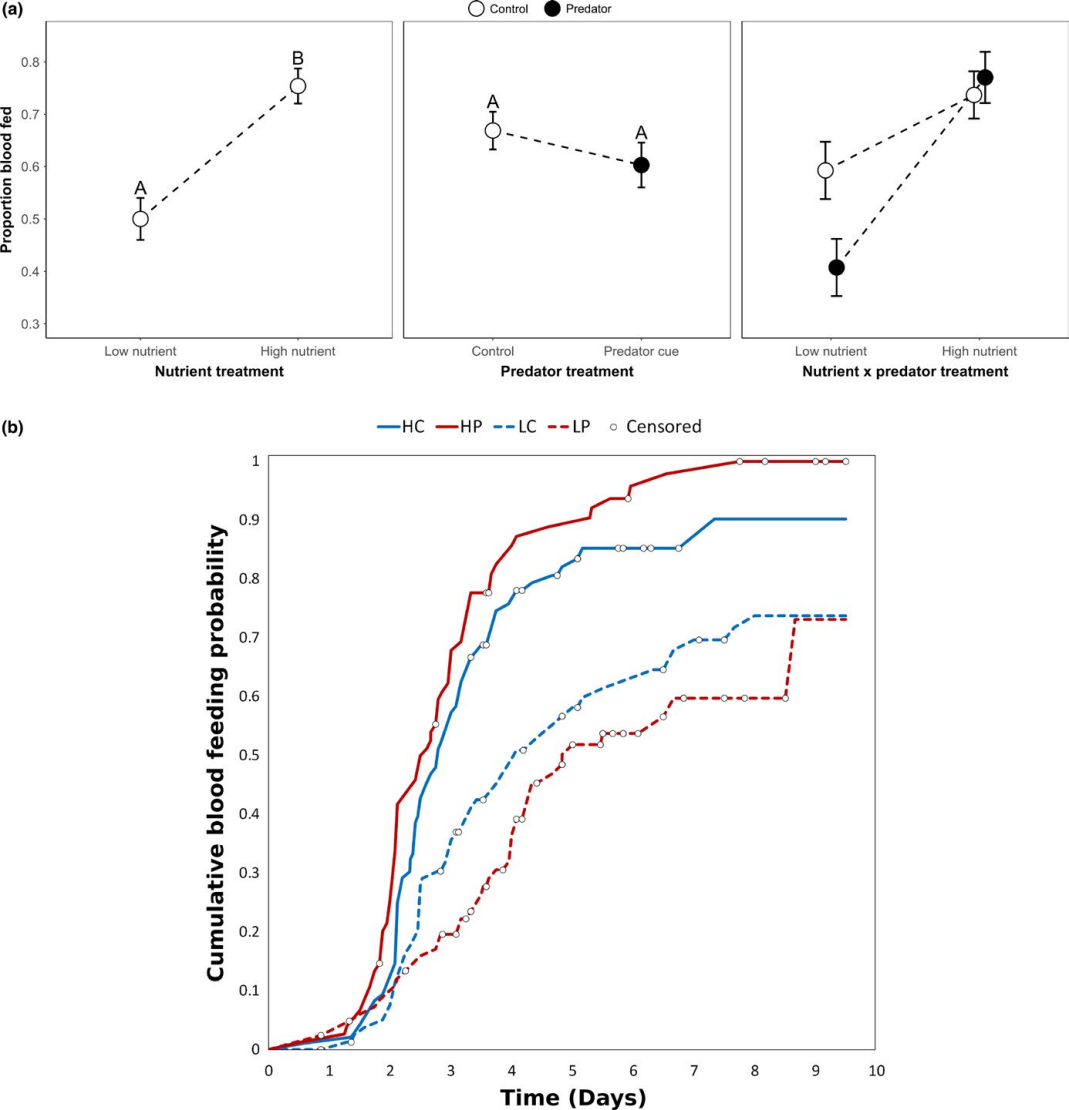
(a) Least squares mean proportion of females that blood fed ± SE was significantly greater in the high versus low nutrient treatment, based on Tukey–Kramer multiple comparisons (indicated by the letters above the means). Pairwise comparisons were not done for the nonsignificant interaction term. (b) Cumulative blood feeding probability. Censored points are females that did not blood feed after being provided with at least 2 blood feeding opportunities

A significantly lower proportion of females blood fed in the low nutrient treatments, (Figure 5a) and there was a marginally nonsignificant interaction between the nutrient and predator treatments (Figure 5a, general linear model: Predator: *F*_1,24_ = 1.39, *p* = 0.25; Nutrient: *F*_1,24_ = 21.53, *p* < 0.0001; Predator × nutrient: *F*_1,24_ = 3.71, *p* = 0.066). The latency of females to blood feed depended upon the interaction between predator cues and nutrient treatments (Figure 5b, Cox proportional hazard analysis: Predator: Wald χ^2^ = 1.773, adjusted *df *= 0.897, *p* = 0.161; Nutrient: Wald χ^2^ = 39.01, adjusted *df *= 0.879, *p* ≤ 0.0001; Predator × Nutrient: Wald χ^2^ = 5.062, adjusted *df *= 0.8719, *p* = 0.020; Container: Wald χ^2^ = 3.22, adjusted *df *= 2.847, *p* = 0.333). Females that were not exposed to predator cues had 1.9 times the hazard of blood feeding if they had high instead of low nutrient availability (i.e., they blood fed more readily). The blood feeding hazard ratio of females exposed to predator cues increased by 3.8 times if they had high instead of low nutrient availability as a larva, whereas the blood feeding hazard ratio for females not exposed to predator cues only increased by 1.4 times if they had high instead of low nutrient availability as larvae.

### 3.6 Fecundity

**Figure 6:**
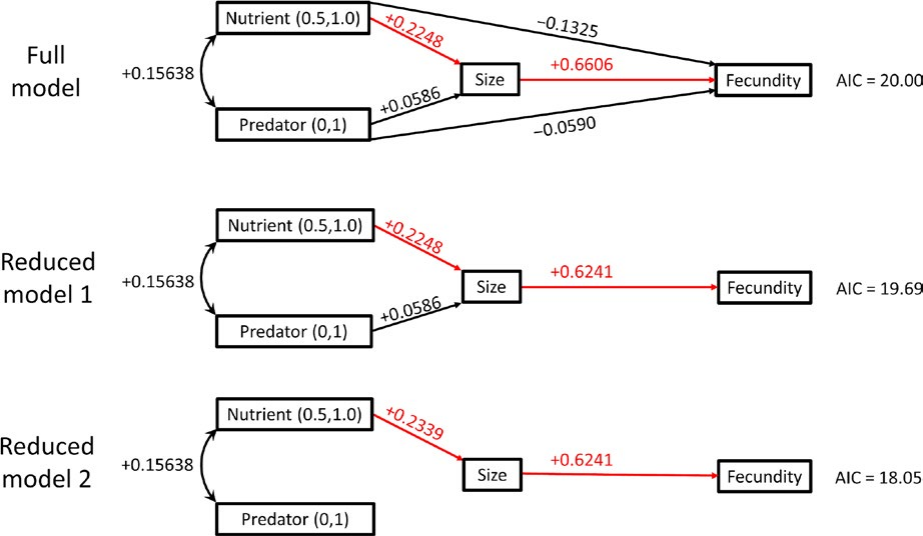
The full model included direct effects between nutrient and predator, direct effects of both nutrient and predator on both female size and fecundity determined by dissection, and a direct effect of female size on fecundity determined by dissection. In each path diagram, significant effects are shown in red. Reduced model 1 eliminated the direct effects of nutrient and predator on fecundity determined by dissection. Reduced model 2 additionally eliminated the direct effect of predator on female size. The best path analysis model was reduced model 2 in which nutrient affected size and size affected number of eggs produced

In the full model of path analysis, greater nutrient availability significantly increased wing length which in turn significantly increased fecundity determined by dissection (Figure 6). There were no significant direct or indirect effects of predator cues on fecundity determined by dissection and no significant direct effect of nutrient availability on fecundity determined by dissection. The best model with the lowest AIC score was the reduced model with no direct effects of nutrient level or predator cues on fecundity determined by dissection and no direct effect of predator cue on size (Figure 6).

### 3.7 Estimated population rate of increase

**Figure 7:**
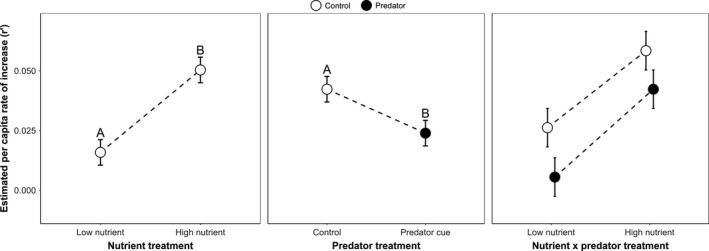
Least squares mean composite index of performance (=estimated per capita rate of increase) ±* SE* was significantly greater in the high versus low nutrient treatment, and for control versus predator treatments based on Tukey–Kramer multiple comparisons (indicated by the letters above the means). Pairwise comparisons were not done for the nonsignificant interaction term

The predator cue and nutrient treatments significantly affected the index of performance *r’ *(which estimates population rate of increase) (Figure 7, ANOVA: Predator: *F*_1,24_ = 5.23, *p* = 0.0314; Nutrient: *F*_1,24_ = 18.38, *p* = 0.0003; Predator × Nutrient: *F*_1,24_ = 0.08, *p* = 0.7798). Estimated population growth rates were significantly greater in cohorts not exposed to predator cues, and cohorts with high nutrient availability.

## 4 DISCUSSION

Nonlethal exposure to chemical cues of predation modified *A. triseriatus* life‐history traits, and these trait‐mediated effects altered our estimates of cohort rate of increase, suggesting effects on population dynamics (Figure 7). Although we did not quantify the behavior of *A. triseriatus*, numerous other studies have shown that larvae respond to alarm cues of predation by adopting low‐risk foraging behaviors (Costanzo et al., 2011; Juliano & Gravel, 2002; Kesavaraju, Alto, Lounibos, & Juliano, 2007; Kesavaraju, Damal, & Juliano, 2007; Kesavaraju & Juliano, 2004, 2010; Wormington & Juliano, 2014). Females in the predator cue treatments had significantly decreased survivorship (Figure 1) and decreased development time under high nutrient conditions (Figure 3). The effect on survivorship is likely the strongest contributor to the decreased the estimated population rate of increase, in part because survivorship has a strong influence on the composite index (Juliano, 1998).

Emergence time of adult females depended on both the predator cue and nutrient treatments (Figure 3). When females were exposed to predator cues, they emerged sooner if they were in the high nutrient treatment. Emerging sooner could decrease female predation risk, but it likely comes at the expense of reduced reproductive fitness due to having a smaller body size, which is positively correlated with fecundity in mosquitoes (Briegel, 1990). In our experiment, adult size was slightly smaller with predator cues than in controls (Figure 2), though this effect was not statistically significant. Predator cues have also been shown to shorten maturation times and reduce the size of other animals with complex life cycles, such as mayflies (Dahl & Peckarsky, 2003; Peckarsky, Cowan, Penton, & Anderson, 1993; Peckarsky & McIntosh, 1998; Scrimgeour & Culp, 1994) and amphibians (Kiesecker, Chivers, Anderson, & Blaustein, 2002; Lardner, 2000; Skelly & Werner, 1990). In our experiment, size of *A. triseriatus* adult females increased significantly in the high nutrient treatments and this in turn indirectly increased fecundity in the first gonotrophic cycle (Figures 2, 6). The absence of a significant effect of predator cues on female size (Figure 2) may have been inadvertently weakened by an increase in nutrient availability arising from the predator cue treatment. Any unconsumed insect biomass from *T. rutilus* predation would decay, increasing bacterial productivity and food availability for *A. triseriatus* larvae in the container (Daugherty et al., 2000; Yee & Juliano, 2006; Harshaw et al., 2007; Yee, Kaufman, & Juliano, 2007). Despite this potential benefit to larvae, adult female size tended to be slightly smaller when exposed to predator cues (Figure 2), suggesting a possible small cost of behavioral and life‐history changes that reduce predation risk.

The composite index we use is only an approximation of the rate of increase and is calculated with the assumption that all eclosing females survive to lay eggs as predicted by the fecundity‐size regression used in the calculation of the index (Livdahl & Sugihara, 1984). Effects of larval environments on adult longevity and on the fecundity‐size relationship must therefore be evaluated independently. Larger adult *A. triseriatus* females survived significantly longer when only provided with water, so any reduction in size from exposure to nonlethal predator cues is expected to decrease survival of adult females when sources of nectar are scarce. The absence of an effect of nutrient on adult longevity is somewhat surprising as nutrient significantly affected adult size, and size significantly affected female longevity with only water. We interpret this combination of effects to mean that for females from the same nutrient treatment, larger size is associated with significantly greater longevity, and conversely for females of the same size, those from high nutrient larval environments have similar longevity to those from low nutrient environments. This pattern suggests that reserves accumulated by females are directly proportional to adult size, regardless of the larval environment.

When adult females were given 20% sucrose, their longevity depended on both predator and nutrient treatments, with females that were from low nutrient conditions and exposed to predator cues having 2.1 times the hazard of death relative to females from low nutrient conditions not exposed to predator cues. Although we detected no significant direct or indirect effects of predator cues on fecundity determined by dissection (Figure 6), predator cues (or their interaction with nutrients) produced a significant reduction in female survival to adulthood (Figure 1) and a small and nonsignificant reduction in adult female size (Figure 2), each of which would have contributed to reducing estimated population rate of increase. Thus, we observe a substantial reduction in *r′ *due to predator cues (nearly 50% reduction—Figure 7). This reduction is likely a substantial underestimate of the life‐history impact of nonlethal effects of predators because the index of performance we used also assumes all individuals have the same average time to acquire a blood meal and that all females live long enough to reproduce once, and only once. Although female longevity without sugar was unaffected by predation cues (Figure 4a), when females had access to sugar, predation cues increased the hazard of death (i.e., shortened adult life) particularly when combined with low nutrient availability (Figure 4b). Further, the combination of low nutrients and predator cues significantly delayed blood feeding (Figures 3 and 5), which is essential for egg production, and this effect would also contribute to a reduction in population rate of increase for a real cohort.

Costanzo et al. (2011) investigated trait‐mediated effects of a different predator on *A. triseriatus* and found significant life‐history costs for males in the form of prolonged larval development times and shortened adult longevity, but found no effects of predator cues on female life‐history traits. We found no effects of predator cues on males, but significant life‐history costs of predator cue exposure for females (decrease in survivorship, significantly faster emergence times when given higher resource levels, significantly reduced adult longevity when given lower resource levels). Male *A. triseriatus* exhibit a more extreme shift in behavior in response to predator cues than do females—particularly when they are well‐fed—increasing their resting behavior at the surface which reduces their predation risk (Wormington & Juliano, 2014). Although female larvae may not change behavior as strongly as do males in response to predator cues (Wormington & Juliano, 2014), females still may face significant life‐history costs from any reduction in foraging due to their higher demand for resources to produce greater adult body size. Other studies on trait‐mediated effects of predation or predation cues on mosquitoes have found retarded larval growth and development, reduced adult size, shortened adult longevity, and reduced teneral reserves of lipid, protein, and carbohydrate (*Culex pipiens*: Beketov & Liess, 2007; *Aedes notoscriptus*: van Uitregt et al., 2012; *Aedes aegypti*: Bellamy & Alto, 2018; Chandrasegaran et al., 2018), and females are usually more strongly affected than males due to greater reproductive costs (Chandrasegaran et al., 2018; Wormington & Juliano, 2014). These nonlethal effects of predators are more likely to be important and detectable when food availability to larvae is limited.

Willingness of *A. triseriatus* females to blood feed depended on both the nutrient level and whether they were exposed to predator cues. Females that were exposed to predator cues were almost 4 times as likely to blood feed if they had higher resource availability as larvae. Females in the low nutrient treatments that were exposed to predator cues were least willing to blood feed possibly because they needed first to increase their energy reserves by consuming sugar. This adds to the growing body of evidence that carry over effects can alter traits other than just adult size and adult fitness (Benard & Fordyce, 2003; De Block & Stoks, 2005). The consequences of these interactive effects of predator cues on blood feeding could be important for vector‐borne disease. However, the complex and multifaceted effect of predation on mosquito life history, and of that life history's effects on disease transmission, render it difficult to predict the effects of any change in a single life‐history variable (like propensity to blood feed) on likelihood of disease transmission. It seems likely that a relatively complex, individual‐based model (Grimm & Railsback, 2005) of larval growth and development and adult life history will be needed to generate predictions of the consequences of predation cues for vector‐borne disease.

The nonlethal effects of predators like *T. rutilus* on *A. triseriatus* larvae could have complex effects on mosquito vectorial capacity (Brady et al., 2016; Smith et al., 2012), which describes the number of secondary infections arising from a single infected individual over the course of their illness in a population that is completely susceptible to the disease (Garrett‐Jones, 1964). Females were most willing to blood feed when they were exposed to predator cues in a high nutrient larval environment (Figure 5) and predator cues produced a nonsignificant trend toward smaller adult size (Figure 2). Both of these results suggest nonlethal effects of predators should increase vectorial capacity, because blood feeding and smaller body size increase the likelihood of viral dissemination to the salivary glands and transmission of La Crosse virus (Bevins, 2008; Grimstad & Haramis, 1984; Grimstad & Walker, 1991; Paulson & Hawley, 1991). However, the reduction in adult body size of females also decreased their survival probability when females were only given access to water, suggesting a greater chance of death before becoming infected and living long enough to transmit the La Crosse virus when nectar sources are scarce. Further, predator cues reduced survivorship to adulthood (Figure 1) and increased development time (Figure 3), both of which should decrease population growth rate (Figure 7). If that decrease results in a smaller adult population, the result is expected to be a decrease in vectorial capacity. Thus, fear during the larval stages could have important, but complex, practical consequences for the health threat posed by this, and perhaps other mosquitoes.

Studies of nonlethal effects of predators across a diverse range of taxa have shown that the trait‐mediated effects of predators can be as strong or stronger than the density‐mediated effects of lethality (Lima, 1998; Werner & Peacor, 2003). The threat of predation can induce changes in prey morphology, life‐history and behavioral traits (Benard, 2004; Wisenden, 2003). The general pattern that has emerged from these studies is that prey respond to predation risk by reducing their activity levels or increasing time spent in refugia, resulting in reduced consumption rates and altered life‐history traits (typically slower growth rates, smaller adult size, reduced fecundity because of that reduced size, reduced longevity) (Lima, 1998; Werner & Peacor, 2003). These effects of predator‐induced fear are widespread taxonomically and ecologically (e.g., Peckarsky et al. 1990; Skelly & Werner, 1990; Thomson, Forsman, Sardà‐Palomera, & Mönkkönen, 2006; Creel, Christianson, Liley, & Winnie, 2007; Creel, Winnie, & Christianson, 2009; Hernández & Laundré, 2005; Beckerman, Uriarte, & Schmitz, 1997). Nonlethal effects of predation cues on larval *A. triseriatus* also have the potential to induce a trophic cascade affecting the microorganisms that are the food of mosquito larvae via predator‐induced changes in mosquito foraging behavior (Albeny‐Simões, Murrell, Vilela, & Juliano, 2015). The nonlethal effects induced by fear of predation can dramatically alter prey behavior and life‐history traits, which can cascade into substantial population, community and ecosystem effects.

## AUTHORS’ CONTRIBUTIONS

S.A.J. conceived the ideas and experimental design. G.D.O. ran the experiment and collected the data. G.D.O and S.A.J. completed the statistical analysis. G.D.O wrote the first draft of the manuscript. Both authors contributed critically to manuscript revisions and approved the final manuscript version prior to submission.

## Data Availability

Data are available from the Dryad repository (Ower & Juliano, 2018): https://doi.org/10.5061/dryad.62940t0
